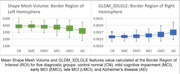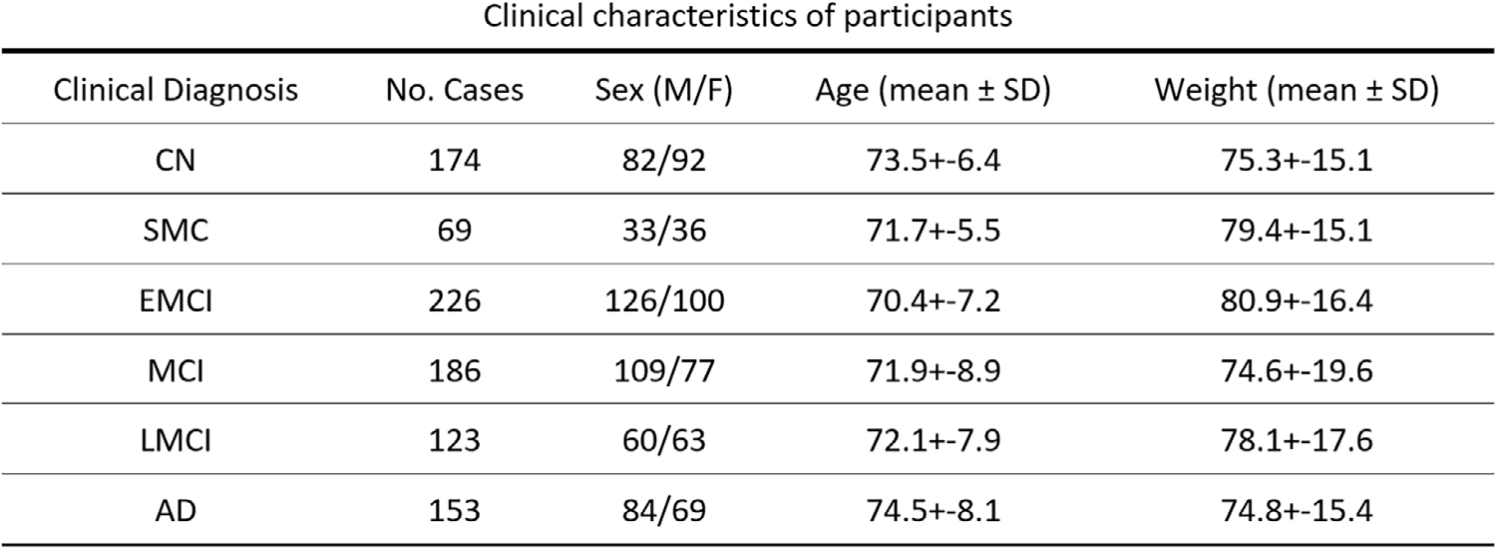# Investigating Alzheimer’s disease progression using a radiomics approach: The hippocampal‐amygdala border in FDG‐PET Scans

**DOI:** 10.1002/alz.094982

**Published:** 2025-01-09

**Authors:** Ramin Rasi, Albert Guvenis

**Affiliations:** ^1^ Bogazici University Institute of Biomedical Engineering, Istanbul, Istanbul Turkey

## Abstract

**Background:**

Alzheimer’s disease (AD) is a progressive neurodegenerative disorder characterized by memory loss and cognitive decline. Detecting AD early and tracking its development is essential for better management. This study delves into the potential of radiomics to identify the AD stages and monitor its progression using FDG PET images. The particular objective is to explore the potential of tracking progression using a reduced and meaningful set of imaging biomarkers at the borders of neighboring brain regions.

**Method:**

Our study analyzed 18FDG‐PET scans from 931 participants across five stages of AD from the ADNI database. Focusing on 95 brain regions, we employed the PyRadiomics tool and eight feature selection techniques. The Hippocampus, Amygdala, and Entorhinal regions emerged as consistently important across these methods. We delineated the connecting regions between these regions using a sliding window approach and identified the best two features that help detect AD by the use of a random forest classifier. We then plotted the values of the two features to track progression.

**Result:**

The connectivity area between the hippocampus and amygdala demonstrated superior efficacy for diagnosing AD progression. The two most important features for this region, namely “Shape Mesh Volume“ and SDLGLE ”GLDM Small Dependence Low Gray Level Emphasis" exhibited the highest classification capability for predicting AD versus control normal individuals (Accuracy = 0.77, sensitivity = 0.73, specificity = 0.81). We plotted these two features across deteriorating stages. The features mean values were able to demonstrate the incremental deterioration. The variance of the feature values increased for deteriorating cases.

**Conclusion:**

We found that the small connection border between the hippocampus and the amygdala can serve as a biomarker to help us partially detect and interpret the presence and severity of AD. In particular, we found that changes in the volume and a measure called SDLGLE within this small border region were strongly linked to AD progression. The SDLGLE increase suggests a potential weakening of the tissue structure, possibly due to neurodegeneration. This preliminary finding highlights the power of a minimal set of radiomic features to not only detect and potentially explain the mechanisms behind AD progression but also to help track disease severity.